# Successful management of multiple-systemic Langerhans cell histiocytosis involving endocrine organs in an adult: A case report and review of literature

**DOI:** 10.1097/MD.0000000000011215

**Published:** 2018-06-29

**Authors:** Junhui Xie, Zhen Li, Yi Tang

**Affiliations:** a Department of Endocrinology; b Department of Radiology; c Department of Hemotology, Tongji Hospital, Tongji Medical College, Huazhong University of Science and Technology, Wuhan, China.

**Keywords:** adult, Langerhans cell histiocytosis (LCH), thyroid

## Abstract

**Rationale:**

Langerhans cell histiocytosis (LCH) involving non-endocrine organs has been frequently reported, whereas LCH involving endocrine organs is rare and the mechanism is unclear.

**Patient concerns:**

We report a case of multiple-systemic Langerhans cell histiocytosis (LCH) that first manifested with thyroid goiter, followed by pituitary and liver involvement.

**Diagnoses:**

The diagnosis was confirmed based on immunohistochemistry of the thyroid and liver.

**Interventions:**

The patient was treated with thyroidectomy combined with chemotherapy and radiation therapy for thyroid and liver, respectively.

**Outcomes:**

Surprisingly, the patient presented with clinical remission and no new lesion of LCH was found during follow-up over 10 years.

**Lessons:**

LCH involving the endocrine system is unusual and easily misdiagnosed or delayed, especially when the thyroid and pituitary glands are involved. Pathological examination is necessary for a definitive diagnosis. Regular examinations, such as anterior and posterior pituitary hormones, should be especially evaluated annually in the patients with LCH involving endocrine system.

## 1 Introduction

Langerhans cell histiocytosis (LCH) is a group of multiple-systemic diseases mainly involving the liver, spleen, lymph nodes, skin, lungs, bone marrow, and endocrine organs. LCH involving nonendocrine organs has been frequently reported, whereas LCH involving endocrine organs is very rare and the mechanism is unclear. It has been reported that adult patients comprise 30% of reported cases, which is less than the number of cases encountered in children.^[1]^ Bone, lung, skin, and posterior pituitary usually are predominately involved in adult cases; however, the liver, spleen, lymph nodes, and bone marrow are more commonly involved in pediatric cases.^[2]^ Different clinical manifestations and prognosis are seen in adults and in children with LCH. Herein, we report one unusual case of an adult patient with LCH involving endocrine organs.

## 2 Case presentation

**Figure 1:**
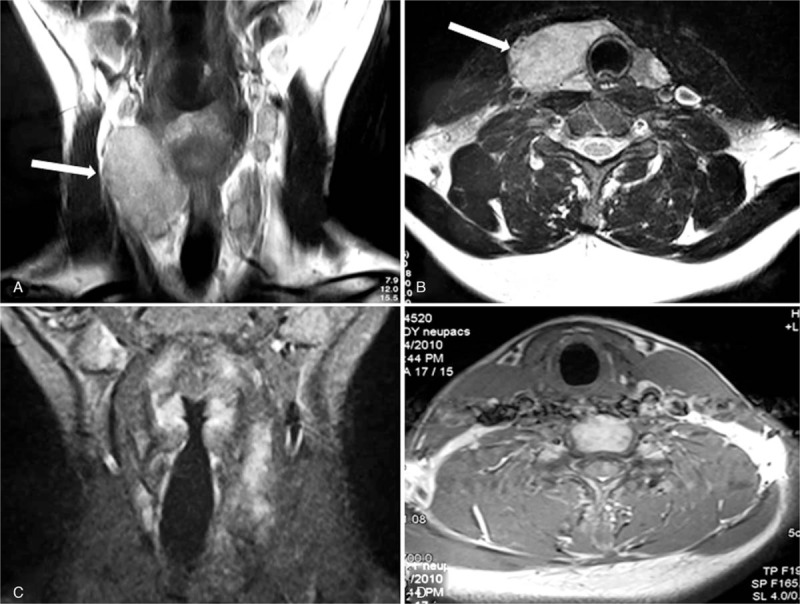
Magnetic resonance image of thyroid showed the obvious enlargement on the right lobe of thyroid in the coronal plane (A) and cross section (B) before surgery; It showed the right lobe of thyroid has no lesions four years (C and D) after surgery.

**Figure 2:**
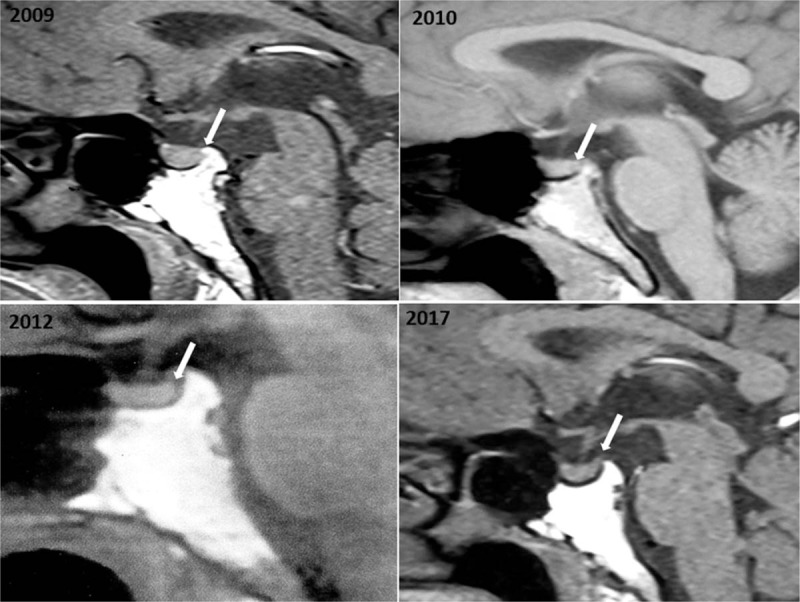
Follow-up magnetic resonance image of pituitary gland in several years. The bright spot was always loss in the posterior of pituitary gland.

A 41-year-old man was accidently detected with a mass without tenderness on the right lobe of the thyroid. B-ultrasound showed that the morphology and echo of thyroid were abnormal though without nodules. Magnetic resonance imaging (MRI) showed significantly enlarged right lobe of the thyroid with a clear boundary and uneven density of mass, partly involving the isthmus of the thyroid, which was suspected as being thyroid adenoma (Fig. 1). The thyroid function testing and emission computed tomography (ECT) scan were normal. Physical examination showed the following: right lobe of the thyroid showed no-fixed II degree swelling with no tender and obvious nodules. The liver and spleen were normal. Bone marrow biopsy was normal and the percentage of eosinophils in peripheral blood was 2.97% (normal range, 0.5%–5%). The patient had a history of hypertension for 7 years and hepatitis for 10 years. There was no family history of thyroid diseases and radiation ray contact. The patient underwent a right thyroid resection and dissection considered the possibility of thyroid adenoma. However, the pathology after surgery indicated LCH because immunohistochemistry staining confirmed CD1a (+), S100 (+), CD68 (+), Valentine (+), and thyroglobulin (+). The thyroid lesion gradually improved after the patient further received radiotherapy of 16 regimens combined with interleukin-2 therapy. The patient regularly monitored the function of thyroid and began to take 50 μg Euthyrox (levothyroxine) per day since being diagnosed with hypothyroidism 3 years after subtotal thyroidectomy. In 2007, the patient was again admitted to our hospital with the complaint of thirst and polyuria for several months. Urine osmolality was obviously lower than blood osmolality and urine specific gravity was less than 1.005. Further, fluid restriction test was positive and MRI of the pituitary revealed loss of the pituitary posterior lobe signal and the pituitary stalk was slightly thicker (Fig. 2). We considered the diagnosis of central diabetes insipidus (DI) and the patient received 3 tablets of desmopressin acetate (0.3 mg/day) with improvement of the symptoms. However, the thyroid and total bone ECT scan were both normal.

**Figure 3:**
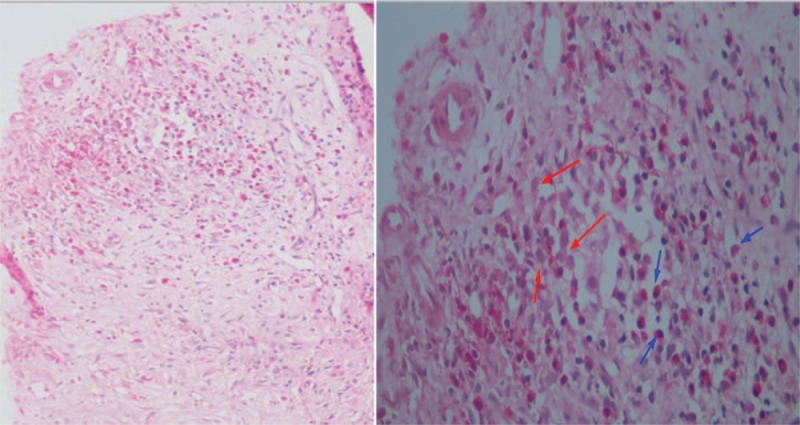
It showed the hepatic immunohistochemistry by percutaneous liver biopsy in the patient. Typical Langerhans cells with weakly eosinophilic cytoplasm and nuclei with nuclear hooves (red arrow) and it showed a large numbers of eosinophilia (blue arrow) in (A) (H&E ×100) and (B) (H&E ×400).

**Figure 4:**
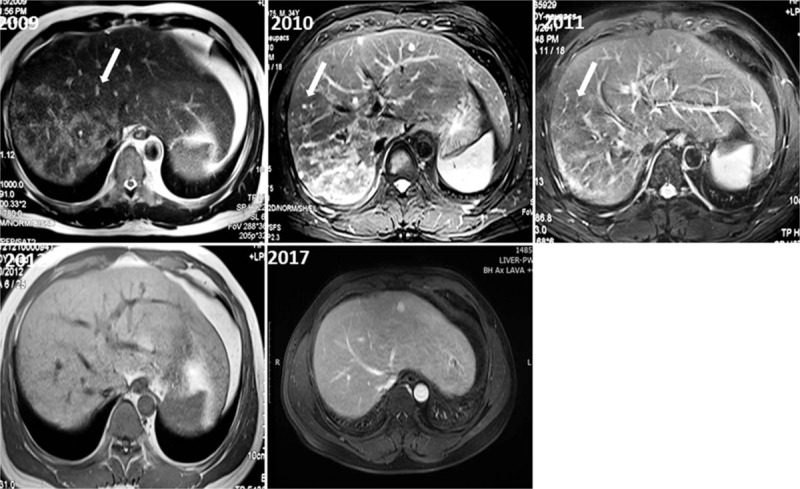
Scatter small high signal intensity was observed in the right lobe liver parenchyma on T2-weighted MR in 2009. The scope of the lesion was reduced obviously in 2010 and it only located in the right posterior lobe. In 2011, only small hyperintensity signal on T2-weighted MR images can be detected. There was no lesion can be find the right lobe during the T1-weighted MR images in 2012 and enhancement MR images in 2017. MR = magnetic resonance.

In 2008, the patient felt pain and distension in the upper abdominal area and was admitted to the hospital. Abdominal computed tomography (CT) showed diffuse fine nodules on the right hepatic with partly fusion. Immunohistochemical staining showed there were Langerhans cells with ovoid to reniform nuclei with grooves by percutaneous liver biopsy (Fig. 3), meanwhile in which the histiocyte-like cells S-100 (+) and CD1a (+) diagnosed as liver Langerhans cell. Liver function testing revealed that alkaline phosphatase (ALP) was 233 U/L (normal range, 40–160 U/L), gamma-glutamyl transaminase was 220 U/L (normal range, <50 U/L) and total bilirubin, liver transaminase and alpha-fetoprotein were in the normal range. Further, the patient received chemotherapy of CHOP regime (CTX1350 mg, VCR2 mg, prednisone 100 mg for 5 days,) and radiation therapy of 11 regimens including 16.5 Gy dose. Surprisingly, we found that the hepatic local region in the patient had remarkably improved and finally was normal during 5 years of follow-up. There was no lesion found in the right lobe during the T1-weighted MR images in 2012 and enhanced MR images of the liver in 2017 (Fig. 4). We considered the patient in clinical remission while the examination of thyroid and pituitary was favorable.

## 3 Discussion

Paul Langerhans first described abnormal proliferative Langerhans cells characterized by irregular shape, unclear border, eosinophilic or mild vacuole, round or oval in the epidermis in 1868. Under the electron microscopic, Langerhans cells showed visible characteristic rod-shaped striped organelles, part of the end of the tennis-like vesicle-like expansion called Birbeck body. The International Histiocyte Society suggested all 3 entities formally identified LCH in 1987: Hand–Schüller–Christian disease, Letterer–Siwe disease, and eosinophilic granuloma of bone. According to the recently developed classification scheme, definitive diagnosis of LCH complied with the expression of the CD1a antigen on the lesion cell surface.

The pathogenesis of LCH is still unclear.^[3,4]^ Whether LCH is a disease of malignancy or immune disorder is controversial. Now tumor and immune dysfunction have become 2 major controversial hypotheses. It has been proven that BRAF gene V600 mutation exists in most cases of the LCH disease, which provides a proper target to cure it. The targeted therapy with the inhibitor of mutated BRAF, vemurafenib, has been used in life-threatening LCH and yielded strikingly efficacious results. However, the tumor specific characteristic has not been confirmed yet, depending on the gene mutation and some clone evolutions.^[4]^

Langerhans cells are located in the epidermis, respiratory and genital epithelia. CD1a is a MHC class I-like protein and is heavily expressed on LCs It is internalized through receptor-mediated endocytosis with langerin and presents the microbial glycolipid antigens to T cells, then initiates the T-cell immune responses. A recent study on the pathogenesis of LCH shows that the improvement in the LCs survival depended more on the regulatory T-cell (Treg) amplifications other than its uncontrolled proliferation. The former leads to the weak control of the LCs in the host immune system. Such a hypothesis creates new direction for LCH therapy and pathogenesis research.

LCH presents with a wide spectrum of clinical manifestations and almost the whole tissue could be involved with or without associated dysfunction. Pathological infiltration of Langerhans cells to tissues or organs is a key factor in the diagnosis of LCH, combined with clinical manifestation and radiology scan. Here, we emphasis on clinical manifestation and advances in treatment of LCH involving endocrine organs in an adult.

### 3.1 Involvement of thyroid

LCH involving the thyroid is very rare and more frequently reported in adults than in children. Usually thyroid enlargement or nodules is the chief complaint by patients. Clinical manifestations were mostly presented with diffuse thyroid enlargement and euthyroid, followed by nodular thyroid enlargement and hyperthyroid and hypothyroid.^[5]^ Patten DK reviewed 75 literatures involving thyroid LCH during the recent 50 years (75% were adults and others were children), 59% of which presented with goiter, 25.8% of which presented with thyroid nodules and 40.9% of which presented with euthyroid, followed by hypothyroid and subclinical hypothyroid.^[6]^ Therefore, the differential diagnosis of LCH involving the thyroid is difficult because the signs and symptoms are variable and nonspecific. Notably, the thyroid nodular or swelling might be misdiagnosed as the following diseases: benign tumors, undifferentiated carcinoma, lymphoma, lymphoid thyroiditis, chronic granulomatous thyroiditis although fine- needle aspiration is essential for LCH.^[7]^ The experts suggested that physicians should consider LCH when cytoplasmic large cells on the background of lymphocytes and eosinophils in thyroid pathology were observed more abundantly.

Once LCH is diagnosed, the whole spectrum of examinations, such as CT scan, bone marrow biopsy, bone ECT, should be performed to determine multisystem involvement. Literature review reported 42.2% of thyroid LCH cases received thyroidectomy, 30.3% of which received surgery and adjuvant chemo-radiotherapy, and 36.4% of which showed the prognosis was favorable with tumor-free follow-up ranging from 3 months to 9 years.^[6]^

### 3.2 Involvement of the hypothalamus and pituitary

Actually, the pituitary presenting with DI is commonly reported in LCH involving the endocrine system. Immunohistochemistry from patients with LCH showed infiltration of a monoclonal population of Langerhans cells. Polyuria and polydipsia are the main manifestations of posterior pituitary dysfunction. However, in most cases the clinic manifestations usually were nonspecific, presented with common complaints: appetite (morbid obesity), thermoregulation (hypothermia), adipsia, sleep, and short-term memory.^[8]^ The retrospective research reviewed 12 LCH adult patients involving DI who were followed-up for a median of 10 years which evaluated the frequency and progression of LCH-related anterior pituitary and other nonendocrine hypothalamic dysfunction.^[8]^ The study showed that 67% of patients developed one or more anterior pituitary hormonal deficiencies at a median of 4.5 years (range, 2–22 years) after the diagnosis of DI, followed by growth hormone (GH) deficiency (8/12), follicle stimulating hormone-luteinizing hormone deficiency (7/12), thyroid-stimulating hormone-adrenocorticotrophic hormone (TSH-ACTH) deficiency (5/12) and panhypopituitarism (5/12). Another study also draw similar conclusions: the incidence of GH deficiency (54% at 10 years) ^[9]^ and neurodegeneration induced by radiotherapy (76% at 5 years) ^[10]^ was high in frequency, followed by gonadotropin deficiency, whereas TSH or corticotropin deficiencies occurred less frequently. The risk for DI in the patients with LCH mostly originated from the research in children, which included multisystem disease and craniofacial involvement, particularly of the ear, eye, and oral region at the time of diagnosis, and had a 4.6-fold relative risk for developing DI.^[10]^ Radiological examinations indicated infiltration of LCH to the hypothalamic pituitary as following: enhancing stalk or hypothalamic lesions and loss of the posterior bright spot.

Literatures suggested small dose of chemotherapy might avoid the progress of disease and involvement of multisystem organs at the initial process of LCH. However, the definitive regimen of chemotherapy was still uncertain. At present, LCH involving the pituitary often is irreversible; both chemotherapy and radiation-therapy could not improve the symptoms of pituitary hormone deficiency. Therefore, regular and prolonged assessment of endocrine hormones and appropriate hormonal replacement should be suggested.

### 3.3 Involvement of other endocrine organs

In addition, other endocrine organs including parathyroid, adrenals, and ovaries were occasionally reported, mostly on the basis of radiological or autopsy findings.^[11]^ It is worth noting that endocrine dysfunctions such as primary hypothyroidism, hyperparathyroidism and even diabetes mellitus are easily ignored in the patients with LCH by physicians.

### 3.4 Involvement of liver

LCH involving the liver is also rare. The infiltration of Langerhans cells to the liver leads to hepatic injury, presents with portal lymphadenopathy, cholestatic hepatomegaly and eventual liver failure. It should be distinguished from sclerosing cholangitis. At present, no favorable medicines can reverse or prevent the progressive biliary fibrosis associated with LCH. In most cases, patients received liver transplantation finally.

No available clinical guideline is suggestive of treatment of LCH. Therefore, many clinical trials have investigated the efficacy and safety of new drugs on LCH. It was indicated that early switch to salvage therapy of the poor responders may significantly decrease mortality and improve survival by 5 years.^[12]^

LCH therapy depends on its risk stratification and lesion location. LCH therapy includes 2 categories according to its lesion location, single system and multisystem. The former is subdivided further into single site and multiple sites. The latter can be subdivided into high-risk and low-risk group according to the involvement of risk organs such as liver, lung, spleen and hematopoietic system. About 80% of multisystem LCH fall in the risk group, with at least one risk organ involved and have poor prognosis. Although the LCH tumor pathogenesis has not been determined, chemotherapy is still an efficient method according to the clinical practices.^[13]^

For the single system therapy, a few reports of reversal of isolated DI with 2-chlorodeoxyadenosine (2-CDA) have been reported.^[14]^ Because of the high incidence of growth hormone deficiency and of radiological neurodegeneration in patients with DI, it was currently recommended to treat recent-onset isolated DI with prolonged low-dose chemotherapy. However, the optimal therapy regimen and the exact duration of therapy have not been defined yet. One research showed that the central nerves system (CNS) mass lesion treatment with 2-CDA caused complete radiographic response in 8 of the 12 LCH patients,^[15]^ and other methods such as intravenous immunoglobulin and trans-retinoic acid or low-dose cytosine arabinoside have been tried, yet the result was unexpected.^[16]^

For the multiple-system LCH therapy, low-risk LCH (without liver, lung, spleen, and bone marrow involvement) should adopt the low-dose and low-frequency therapy in order to prevent DI and other late complications. On the contrary, higher risk LCH should adopt more prolonged and intensive combination chemotherapy such as prednisone, etoposide, vinblastine, 6-mercaptopurine and methotrexate for 12 months in order to improve the long-term survival.^[17]^ It is appropriate and efficient for these patients to choose the chemotherapy because their liver and hyperthyroid infiltration classified them as belonging to the risk stratification. With 2 large prospective multicenter trials comparisons, the 12 months of combination chemotherapy is beneficial for the risk LCH patients. A recently published Japanese study showed that the early switch to the salvage therapy by the poor responders may significantly decrease mortality and the overall survival rate by 5 years in about 95% of the MS-LCH patients.^[12]^

For the refractory multiple-system LCH patients who fail to respond after 2 courses of therapy, no effective salvage regimen was suggested. Although a lot of combination regimens have been tried, it was pity that none of these combinations appear to improve survival, nor have they shown other benefits.^[18]^

Cladribine has given durable responses in patients with low-risk disease reactivation. For patients with high-risk refractory disease, a more intensive regimen with higher doses of 2-CDA in combination with cytarabine has been effective.^[19]^ The combination of asparaginase with Ara-C has also been found to be useful in some patients with multiple relapsed LCH. And more recently clofarabine, a new generation of deoxyadenosine analog, intermediate-dose methotrexate (100–175 mg/m^2^), thalidomide, tumor necrosis factor and its inhibitor, cyclosporine-A and stem cell transplantation all have been effective in the refractory/relapsed LCH, and organ transplantation is an effective therapy for end-stage liver and lung chronic fibrosis (sclerosing cholangitis) in the absence of active LCH.

In summary, LCH involving the endocrine system is relatively rare, especially at first presentation with thyroid, followed by pituitary and liver involvement. Although the principle of management is due to a lack of universally accepted guidelines, it was suggested that resection of subtotal, nearly total or total thyroidectomy was the preferred treatment for LCH localized to the thyroid. Perhaps LCH in adults run a relatively indolent course and a more conservative approach may be required.

There are several characteristics of our case such as the following: The LCH case presented with rare endocrine system involvement, not with the common organs normally seen in male adults. Thyroid enlargement was the first main symptom, followed by other multi-system symptoms. The patient quickly received sublobe thyroidectomy combined with radiation therapy. Diagnosis of LCH involving the liver was performed by fine needle aspiration. The symptoms were gradually relieved with chemotherapy and radiotherapy during the first 5 years of follow-up.

The review of literature suggested surgery was the priority therapy for the LCH involving the thyroid. However, definitive treatment with thyroidectomy in LCH is still controversial due to the lack of prospective randomized studies. In one study, 30.3% of reported cases received thyroidectomy combined with chemotherapy and radiation-therapy.^[6]^ Therefore, we considered the combination management on the early process when LCH was localized to one organ which could improve the prognosis of LCH.

## 4 Conclusions

In conclusion, LCH involving the thyroid is unusual and is easily misdiagnosed or delayed. The prognosis of LCH involving the liver is relatively favorable. Studies on LCH with the emphasis on common organs such as pulmonary, liver, bone and skin in nonendocrine system are required. Regular examinations such as that of the anterior and posterior pituitary hormone should be especially evaluated annually in the patients with LCH involving endocrine system. This patient belonged to the high-risk MS-LCH because of the consequent involvement of the thyroid gland, pituitary and liver; therefore, the combination with chemotherapy was the favorable choice. The effect of treatment also showed the complete remission of liver radiology check. However, the further 12 months prolonged chemotherapy should be considered to prevent late complications and improve longer survival.

## Acknowledgments

We thank all the participants who took part in the studies described in this article.

## Author contributions

Dr. JHX was involved in acquisition and investigation of data, funding acquisition , drafting and reviewing the manuscript. Dr. ZL described and wrote the figure legends of the manuscript. Dr. YT participated in critically writing and revising the manuscript. All authors read and approved the final manuscript.

**Conceptualization:** Junhui Xie.

**Data curation:** Junhui Xie.

**Formal analysis:** Junhui Xie.

**Funding acquisition:** Junhui Xie.

**Investigation:** Junhui Xie.

**Methodology:** Junhui Xie.

**Project administration:** Junhui Xie.

**Supervision:** Yi Tang.

**Writing – original draft:** Junhui Xie, Zhen Li, Yi Tang.

**Writing – review & editing:** Junhui Xie, Yi Tang.
